# Increasing palliative care capacity in primary care: study protocol of a cluster randomized controlled trial of the CAPACITI training program

**DOI:** 10.1186/s12904-022-01124-x

**Published:** 2023-01-05

**Authors:** Hsien Seow, Daryl Bainbridge, Samantha Winemaker, Kelli Stajduhar, Gregory Pond, Kathy Kortes-Miller, Denise Marshall, Frances Kilbertus, Jeff Myers, Leah Steinberg, Nadia Incardona, Oren Levine, Jose Pereira

**Affiliations:** 1 Department of Oncology, McMaster University, Hamilton, ON Canada; 2 School of Nursing, University of Victoria, Victoria, BC Canada; 3 Department of Social Work, Lakehead University, Thunder Bay, ON Canada; 4 Department of Family Medicine, McMaster University, Hamilton, ON Canada; 5 Northern Ontario School of Medicine University, Thunder Bay, ON Canada; 6 Division of Palliative Care, University of Toronto, Toronto, ON Canada; 7 Department of Family and Community Medicine, University of Toronto, Toronto, ON Canada

**Keywords:** Palliative Care, Education, Health Personnel, Training Programs, Professional Competence, Cluster Randomized Controlled Trial

## Abstract

**Background:**

Primary care providers play a critical role in providing early palliative care to their patients. Despite the availability of clinical education on best practices in palliative care, primary care providers often lack practical guidance to help them operationalize this approach in practice. CAPACITI is a virtual training program aimed at providing practical tips, strategies, and action plans to provide an early palliative approach to care. The entire program consists of 12 sessions (1 h each), divided evenly across three modules: (1) Identify and Assess; (2) Enhance Communication Skills; (3) Coordinate for Ongoing Care. We report the protocol for our planned evaluation of CAPACITI on its effectiveness in helping primary care providers increase their identification of patients requiring a palliative approach to care and to strengthen other core competencies.

**Methods:**

A cluster randomized controlled trial evaluating two modes of CAPACITI program delivery: 1) self-directed learning, consisting of online access to program materials; and 2) facilitated learning, which also includes live webinars where the online materials are presented and discussed. The primary outcomes are 1) percent of patients identified as requiring palliative care (PC), 2) timing of first initiation of PC, and self-reported PC competency (EPCS tool). Secondary outcomes include self-reported confidence in PC, practice change, and team collaboration (AITCS-II tool), as well as qualitative interviews. Covariates that will be examined are readiness for change (ORCA tool), learning preference, and team size.

Primary care teams representing interdisciplinary providers, including physicians, nurse practitioners, registered nurses, care coordinators, and allied health professionals will be recruited from across Canada. The completion of all three modules is expected to take participating teams a total of six months.

**Discussion:**

CAPACITI is a national trial aimed at behavior change in primary care providers. This research will help inform future palliative care educational initiatives for generalist health care providers. Specifically, our findings will examine the effectiveness of the two models of education delivery and the participant experience associated with each modality.

**Trial registration:**

ClinicalTrials.gov NCT05120154.

**Supplementary Information:**

The online version contains supplementary material available at 10.1186/s12904-022-01124-x.

## Background

Several systematic reviews have found that a palliative approach to care can be effectively delivered by primary care providers (PCP) working in community-based interprofessional teams to improve patient and family outcomes [1, 2]. Considering longitudinal relationships and continuity of care, primary care providers are ideally positioned to identify the need for palliative care and to initiate it early in the trajectory of serious chronic or terminal illness among their patients [3]. Research shows PCPs are willing to provide palliative care [4, 5] but often cite the lack of knowledge, confidence, tools, and practical supports to operationalize this approach in practice [6, 7]. Comprehensive palliative care education programs combined with appropriate supports play a critical role in addressing these gaps.

One exemplar education program is the Gold Standards Framework (GSF) Training Program in the United Kingdom. Beginning in 2003, GSF has spread across the country, training over 20,000 providers in 3,500 teams in primary care, long-term care, and hospitals across the country [8–10]. A review of 15 publications evaluating the Gold Standards Framework found the program led to more patients identified on a palliative care registry, earlier access to palliative care, strengthened interprofessional coordination and increased family caregiver satisfaction [8]. Knowledge translation science underscores how its success in achieving outcomes and spreading widely is, in part, related to the program’s emphasis on applied knowledge, skills, and tools that are adaptable and tailored to the local context [11–13]. For instance, the UK has palliative care patient registries via widespread electronic medical records, and financial incentives for reaching targets in the registry.

In Canada, where financial incentives and electronic registries for palliative care do not exist, Pallium Canada has been the national leader for palliative care education since 2000. For instance, between 2015–2019, over 1,600 Pallium Learning Essential Approaches to Palliative care (LEAP) courses were taught across Canada to nearly 30,000 interprofessional health care providers [14, 15]. LEAP has been shown to be effective across multiple domains of palliative care competencies [16, 17]. Pilot studies in cancer and primary care settings have shown that LEAP education combined with practice supports, such as an integrated care model, can increase provider confidence and access to primary-level palliative care [18, 19]. Other practice supports, such as commitment-to-change strategies, have been shown to enable learners to apply knowledge into practice [20].

Further research on effective strategies to optimize the integration of knowledge into practice and behavior change would be beneficial [21]. In particular, research exploring virtual education programs are needed, since this delivery method is becoming more acceptable, even essential in the context of the COVID-19 pandemic [22], and can address some geographic and access issues. Moreover, since self-directed virtual education is becoming more common, rigorous research on the benefits of delivering case-based discussions, expert guidance, and facilitation online to support practice change is needed to address knowledge translation gaps.

### Overview of CAPACITI and Pilot Study

Our study team developed CAPACITI, which stands for the Community Access to PAlliative Care via Interprofessional Teams Improvement program, to provide PCPs with practical skills for better incorporating a palliative approach to care into practice. CAPACITI is a virtual training program aimed at providing practical tips, strategies, and action plans to help primary care teams operationalize and deliver an early palliative care approach to patients with life-limiting illnesses. Our recently completed pilot study of CAPACITI with 22 teams across Ontario [23], demonstrated the feasibility and potential efficacy of this intervention in a pre/post evaluation. Based on the quantitative and qualitative findings from the pilot study, we refined the format and content of CAPACITI and designed this cRCT for robust evaluation. This randomized trial examines the benefits of self-directed education alone versus education plus facilitation using case-based discussions to support local adaptation and practice change.

### Aims

We intend to conduct a randomized controlled trial of a refined version of CAPACITI to examine the impact of this training intervention more empirically on generalist providers. In this article, we present the design and procedures of the CAPACITI trial. The intervention and study implementation process will be described. The intervention will be a facilitated model of CAPACITI training education, and the comparator will be the same materials provided in a self-directed, non-facilitated format. The key aim of the study will be to assess the impact of the Facilitated versus Self-directed versions of CAPACITI on patient identification, core competencies, change in practice, and team collaboration, towards providing a palliative approach to care. Our hypothesis is that the facilitated format will be more effective on all outcomes.

## Methods

### Study design

We are conducting a prospective cluster randomized controlled trial (cRCT) in which the clusters are primary care practices (teams) randomized to either a Facilitated or Self-Directed model of CAPACITI. This program consists of three distinct education modules, each which will be evaluated separately. The primary comparison will be between the trial arms upon completion of a module to compare effectiveness of the two approaches. Our study will also measure the change in outcomes within the same team, before and after completion of a module. The anticipated completion date of the cRCT is July 2023. Ethical approval for this study was obtained from the Hamilton Integrated Research Ethics Board (#13867) (see https://clinicaltrials.gov/ct2/show/NCT05120154 for approved operating protocol).

### CAPACITI Intervention

The development of CAPACITI has been described previously [23]. This intervention originated from an extensive review of existing palliative care training programs and input from national and international experts. CAPACITI differs from other educational interventions in that it is intended to complement established programs that teach core palliative care skills by focusing on the application of these skills in practice by generalists using facilitated, case-based, virtual education. The current iteration of CAPACITI was revised based on the feedback of the pilot study, for example, additional case examples, more concise content, and shorter program duration. In this intervention, participants will enroll in three distinct modules, taken in order; each module is comprised of four sessions (1 h long).

**Fig. 1:**
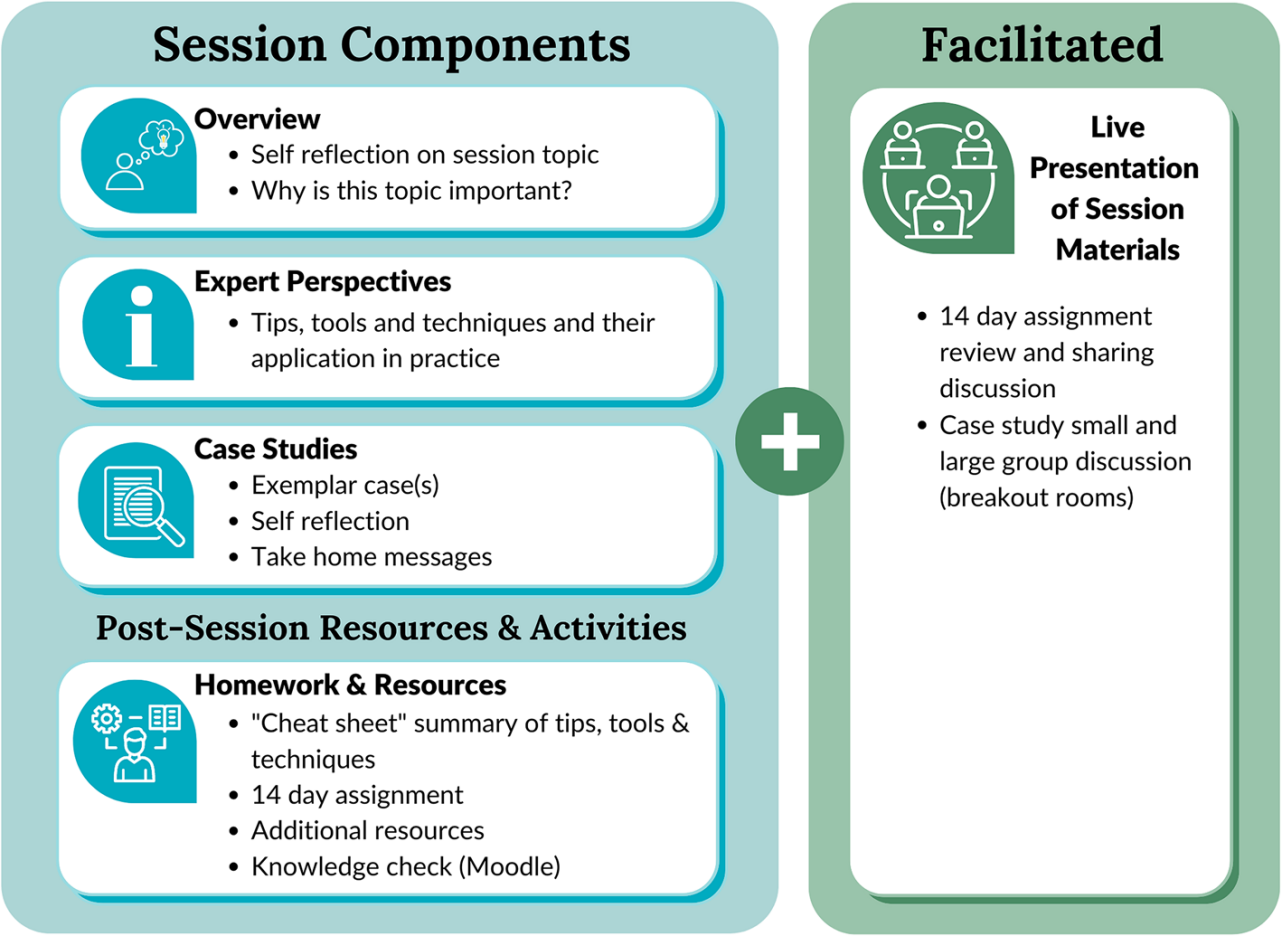
CAPACITI virtual session components

Each module addresses a critical component of implementing a palliative approach to care into primary care practice: (1) Identify and Assess; (2) Enhance Communication Skills; (3) Coordinate for Ongoing Care (including involvement of family and specialists). Over bi-monthly sessions, each CAPACITI module integrates 3 components: clinical education in the form of expert advice and tips; evidence-based tools; and case studies to serve as practical examples. Between sessions, PCPs are to complete an activity to encourage them to apply the session content in practice. The session content of each module is presented in Table 1 and the learning components in Fig. 1. CAPACITI will be provided virtually to participants, hosted on an online learning management system (Moodle, https://moodle.org/). Emails will be sent from the learning management system to participants to notify /remind them of module /session dates, assignment completion, etc.

**Table 1:** Session content of CAPACITI education modules

**Module 1: Identify & Assess**	**Session Objectives**	**Tools**	**14-Day Challenges**
**Session 1**	• Identify expert tips and triggers in the illness trajectory to initiate palliative care upstream	• Supportive and Palliative Care Indicators Tool (SPICT) • Palliative Performance Scale (PPS) tool • Prognostic Indicator Guidance (PIG) tool	• Reflect on how many of your patients would benefit from receiving a palliative care approach
**Session 2**	• Assess practices and strategies to identify, track and monitor patients using a registry	• Palliative Care registry examples	• Start a ‘registry’ to identify and track patients who need a palliative care approach using EMR or non-EMR strategies • Think about who will maintain this registry and flag patients
**Session 3**	• Role model best phrases and practices of how to incorporate assessment skills and strategies in simple ways	• Introduction phrases to introduce a palliative care approach	• Use the everyday language to introduce “assessment” to a few patients
**Session 4**	• Advanced and systematic assessment using evidence-based tools for functional decline and prognosis, distress and complex symptoms	• Clinical Frailty Scale • Distress Thermometer • Comprehensive Problem and Symptom Screening (COMPASS) • Edmonton Symptom Assessment System (ESAS) • Canadian Problem Checklist	• Use the “cheat sheet” to complete a palliative care needs assessment on a patient
**Module 2: Enhancing Communication Skills**	**Session Objectives**	**Tools**	**14-Day Challenges**
**Session 1**	• Improving conversation skills regarding serious illness using everyday language	• Person-Centred Decision-Making: Quick Reference Guide • Model of Person-Centered Conversation, tips and conversation starters	Watch Mr. Young Case and reflect on: 1. What did the patient/caregiver wish for, worry and wonder about? 2. What questions were most important for the clinician to answer for the patient/caregiver? 3. Think about one of your patients: a) Do you know what their goals of care are? b) What plan would you make with your patient for their future? c) What questions could you anticipate and how would you answer those questions? d) What did you do to handle the conversation?
**Session 2**	• Describe ways to explore the patient’s illness understanding and provide illness education in ways that are simple, natural and acceptable to patients	• Same as Session 1	• In the next 2 weeks, think about a few patients in your practice that you could, in the future, use these conversation skills with. Write their names down for now
**Session 3**	• Describe ways to weave-in Advanced Care Planning and goals of care discussions, and explore patients’ goals and values in ways that feel natural	• Model of Person-Centered Conversation • PHRASE TOOLKIT for Conversation and Advanced Illness	• Watch Mr. H Case and reflect on: 1. What did the patient/caregiver wish for, worry and wonder about? 2. What questions were most important for the physician to answer for the patient/caregiver? 3. Think about one of your patients: a) Do you know what their goals of care are? b) What plan would you make with your patient for their future? c) What questions could you anticipate and how would you answer those questions? d) What did you do to handle the conversation? • In the next 14 days, use the cheat sheet and tool kit to have a conversation with one of the patients whose name you wrote down at the end of Session 2
**Session 4**	• Recognize components of successful patient conversations • Apply the model of person-centred conversation to your practice • Use additional tips and strategies to facilitate conversations with patients about serious illness	• Same as Session 3	• Keep practicing these conversation skills • In the next 14 days, keep practicing these conversation skills by having another conversation with a patient in your practice
**Module 3: Enhancing Skills for Ongoing Care**	**Session Objectives**	**Tools**	**14-Day Challenges**
**Session 1**	• Incorporate strategies to allow for proactive care planning in your practice • Establish your role in providing continuity of care for your patients	• Care Planning Strategy table	• Connect with the 1 patient you identified to be proactive and to renew your vows with that patient
**Session 2**	• Leverage and identify your external network of local community resources for support	• Contact List Template • Models of Care Worksheet • Disease Trajectory Diagram	• Using the cheat sheet template, create your own contact list (for you and your team) for palliative care planning • Examples of contact list always had these roles: Physician/NP/RN/RPN • Other roles vary by region. Here are some to consider: Admin, SW, RD, LHIN Coord, PSW, HC, OT/PT, PallCare Consult Coordinator, Hospice, Spiritual, EMS, Hospital, Transition Coordinator, Oncologist/other specialist, Palliative Care Specialist, Volunteers
**Session 3**	• Describe when to interact with specialists and strategies on how to successfully engage specialists in the care of your patients	• External use emergency contact list/phone tree • Communication tips and phrases that you can use when consulting with a specialist	• Talk to a specialist (could be palliative care specialist or other “ologist”) • Use sayings from the cheat sheet to help guide your conversation
**Session 4**	• Identify ways to involve the caregiver in your patients care and how to assess the needs of the caregiver	• Tips on engaging the caregiver	• Explore how the caregiver can be part of the care team • Assess the needs of a few caregivers (use the cheat sheet to help you) • Set up office processes/scheduling appointments for assessing needs of caregivers, including bereavement

### Study groups

The control group will receive access to the online session slide decks, tools, “cheat sheets”, resources, and assignments. The intervention group will receive the same access to the online session materials but will also be invited to participate in facilitated virtual webinars, that includes a presentation of the session slide deck and open discussion of the content (Fig. 1).

- *Control group:* Self-Directed – Access to online CAPACITI materials only (no live presented sessions)
- *Intervention group:* Facilitated – Access to online CAPACITI materials plus each session is presented on a live interactive video conference, including group discussion of the material for adaptation to local context.

We hypothesize that the interactive sessions and opportunity for open discussion offered to the facilitated group in CAPACITI will better assist them to overcome challenges to knowledge translation and implementation, compared to the self-directed group.

### Outcomes

Study outcomes and corresponding measures, summarized in Table 2, are as follows:

**Table 2:** Summary of study outcomes and measures by framework domain

**Data Outcome**	**Kirkpatrick Model Domain Assessed**	**Outcome Measure/Instrument**
**Primary**		
1. Palliative care access and timing	**Results**	Access: Total case load, # identified PC, Timing: Typical timing of when first palliative care will be initiated for cancer and noncancer patients
2. Palliative care competency	**Learning**	End-of-life Professional Caregiver Survey (EPCS) – 20 items
3. Assignment completion / Change in practice	**Learning** **Behavior** **Results**	Checklist of completion of module specific assignments (study created survey) Change survey – 4 items
**Secondary**		
1. CAPACITI confidence	**Behavior**	CAPACITI Competencies Survey – 20 items
2. Team interprofessional collaboration	**Behavior**	Assessment of Interprofessional Team Collaboration Scale II (AITCS II) – 23 items
3. Satisfaction with program	**Reaction**	Perception of webinar content Self-report poll – 4 items
4. CAPACITI effectiveness by context	**Reaction** **Learning** **Behavior**	Semi-structured focus groups to access team perceptions of the CAPACITI program
5. Program analytics	**Reaction**	Learning management system (Moodle) metric tracking (# participants, module completion, time spent in session, downloads)
6. Effectiveness on above outcomes by co-variates	**Impact on all domains**	Team Registration Form (location, electronic medical record platform, etc.) Team Member Registration Member Form (profession, learning preference, palliative care training, years in role, etc.) Organizational Readiness to Change Assessment survey (ORCA) – 31 items

#### Primary outcomes

- 1. Palliative care access and timing, measured based on self-reported: i) number of patients in caseload and number (calculated %) reported as Identified as requiring a palliative approach to care in last 3 months, ii) Typical timing of when PCP initiates a palliative approach to care for their cancer and non-cancer patients, respectively.
- 2. Palliative care competency, measured by scores on the End-of-life Professional Caregiver Survey (EPCS). The EPCS is a 28-item scale developed to assess palliative care-specific educational needs within an interprofessional team related to three main subdomains: Effective Care Delivery (ECD 8-items); Patient and Family-Centered Communication (PFCC 12-items); and Cultural and Ethical Values (CEV 8-items) [24]. Each item is scored on a 5-point Likert scale ranging from 1 (lowest level of skill) to 5 (greatest level of skill). Items represent care-provider comfort with a variety of situations related to palliative and end-of-life care. The EPCS covers all eight domains of the national palliative care guidelines and core lessons of physician-specific and nurse-specific end-of-life education curricula in the USA. The EPCS exhibits strong internal consistency (alpha = 0.96). For the purposes of this study, we will exclude the CEV sub-domain items from the EPCS.
- 3. Assignment completion and perceived change in practice, measured by number of module assignments attempted/completed (checklist) and reported change in thinking, behaviour, processes, and patient/family experience (Assignment Completion & Change Survey). 
- This survey is a two-part, study-created questionnaire based on the CAPACITI module activities. Part A is unique to each module, asking participants to indicate the extent to which they were able to complete each of the session assignments for the module. Response options are: Have not started (1), Started but not completed (2), Completed (3). Part B contains four items assessing changes in thinking, behaviour, processes, and patient/family experience, respectively.

#### Secondary outcomes

- 1. CAPACITI confidence in palliative care, where primary care team member’s capacity is measured by scores on the CAPACITI Competency Survey [23]. The CAPACITI Competency Survey is a study created questionnaire based on the CanMEDS framework for improving patient care by enhancing physician training and the topics covered in the CAPACITI program. CanMEDS, developed by the Royal College of Physicians, delineates critical competencies to effectively meeting the health care needs of patients, including communication, expertise, collaboration, advocacy, and commitment [25] Each item on the Competency Survey is scored on a 7-point Likert scale ranging from 1 (lowest level of confidence) to 7 (greatest level of confidence). The survey was developed and tested in the CAPACITI pilot study, and exhibits strong internal consistency (alpha = 0.96) [23].
- 2. Team interprofessional collaboration, measured by scores on the Assessment of Interprofessional Team Collaboration Scale II (AITCS II) [26]. The AITCS is an instrument designed to measure interprofessional collaboration among team members. The AITCS consists of 23 items considered characteristic of interprofessional collaboration (how team works and acts). Scale items represent three elements considered to be key to collaborative practice. These subscales are: Partnership (8 items), Cooperation (8 items), and Coordination (7 items). Each item is scored on a 5-point Likert scale indicating the extent to which the team exhibits each, ranging from 1 (Never) to 5 (Always). Internal consistency estimates for reliability of each subscale range from 0.80 to 0.97, with an overall reliability of 0.98.
- 3. Satisfaction with CAPACITI program measured by team members’ Session evaluations (poll survey of 4 items).
- 4. Effectiveness and adaptations by local context, focus groups to assess teams’ perceptions of the program overall and impact on practice.
- 5. Program analytics, for each team member measured through the Learning Management System (module pages accessed, time spend on platform, quizzes completed, session attendance).
- 6. Effectiveness on above outcomes by co-variates, contextual factors impacting effectiveness of program outcomes, specifically across self-reported: i) Team/member characteristics (profession, role, palliative care training, years working with team, remuneration model, team size, location), ii) Individual’s level of readiness, measured by scores on the Organizational Readiness to Change Assessment survey (ORCA) [27], and iii) Individual’s preferred learning style (self-directed or group facilitated).

The ORCA measures organizational readiness to implement evidence-based practices in clinical settings. The survey was developed from the Promoting Action on Research Implementation in Health Services (PARIHS) framework, a theoretical model to guide implementation of evidence-based interventions, which contains 3 major domains of evidence, context, and facilitation [28]. The ORCA is intended to be modified to ensure applicability to the intervention being assessed – the modified version for our study contains a total of 31 items with 8 subscales [27].

### Data collection

All survey data will be collected online, self-reported through the learning management system. Module surveys will be completed by PCP participants from all teams at five time points: before (T1) and after Module 1 (T2), after Module 2 (T3) and Module 3 (T4), and at 6 months following final module completion (T5). For Module 2 and Module 3, the post module survey from the immediately preceding module will serve as the baseline survey. Completion of the baseline measures will be a prerequisite to enrollment in CAPACITI. We will follow the Dillman Tailored Design Method to administer the questionnaire with up to five follow-up emails to non-responders [29].

We will conduct virtual focus groups (30 to 60 min in length) approximately three weeks post module completion with a purposive sample of teams from intervention and control groups: 6 to 10 teams in each arm per module (12 to 20 teams total, representing 60 to 100 team members total). The focus group discussion guide was developed and tested in the CAPACITI pilot [23]. In the focus group we will inquire if implementation was perceived as successful (If so, how? If not, why?) and what were the barriers and facilitators. These data will be supplemented by field notes maintained by staff during the study.

### Statistical power /sample size

Sample size calculation is based on the assessment of the primary outcomes of Effective Care Delivery (ECD 8-items) subdomain on the End-of-life Professional Caregiver Survey (EPCS) and the palliative care identification variable (% of patients identified as requiring a palliative approach to care).

Previous work using the EPCS with nurses, physicians, and social workers identified a mean score of 3.6 for the ECD subdomain and a standard deviation (SD) of 1.0 (scale from 1 to 5) [24]. We assumed that a difference of 0.5 in SD (i.e., a delta of 0.5 or a half point on the scale) between treatment groups at T2 would be important to detect. Accounting for the cluster design, we estimate that the correlation between providers within teams was 0.15 and that each team would have a minimum of 4 members participate. Given a two-sided alpha of 0.05, a power of 80%, 192 providers from 48 teams would be required. This will also allow for detection of a 1-point difference increase of the % of patients identified as requiring a palliative approach to care (SD = 2).

We anticipate 100 PCP teams with an average of 4 members per team, in each module. The teams will be from across Canada and geographically diverse (e.g., rural, urban, and remote), which is critical to generating evidence on generalizability in diverse communities across the country.

### Recruitment

Study participants will be members of PCP teams that enroll in CAPACITI (See Appendix for CAPACITI information sheet). Participants can also sign up as individuals or solo providers. Potential teams across Canada will be informed about CAPACITI through a promotional campaign, including direct solicitation and advertising by our partner stakeholders and organizations, including Pallium Canada, Hospice Palliative Care Ontario, Saint Elizabeth Health Care, Canadian Hospice Palliative Care Association, and provincial professional associations, e.g., Medical Association of Ontario. A team wishing to participate in the program will complete a registration form, indicating the team members that will be participating. Team members will then register individually on the Moodle learning management system to enrol in the program.

### Eligibility

To be eligible the “team” or individual must be community-based and willing to provide palliative care to their patients, defined as managing symptoms, addressing psychosocial needs, educating patients and families, and coordinating care. Teams need to include at least one prescribing clinician (e.g., primary care physician, nurse practitioner). Participates in CAPACITI can include physicians, nurses, social workers, office assistants, patient coordinators, etc. The program will be offered free to teams, with the understanding that those participating will complete the educational and data collection components.

Participants will be strongly encouraged to complete an interprofessional, standardized, evidence-based, education program, namely Pallium Canada’s LEAP course (Learning Essential Approaches to Palliative care), prior to starting CAPACITI. LEAP is the most widely recognized palliative care education program for generalist health care providers in Canada [14, 15, 20]. Some topics include the palliative care philosophy and complex management for common symptoms such as pain, delirium, constipation, depression, grief, etc. The courses are taught in-person, or via fully online or hybrid delivery, by certified program facilitators [16]. Studies have shown that the LEAP courses provide primary health care teams with foundational skills that allow them to integrate primary palliative care in their clinics [18].

### Randomization

The unit of randomization is the team: individuals clustered within teams or the individual themselves as their own team. Teams who register will be randomized to either the intervention or control arm using a permuted block design to ensure groups of equal sizes [30]. Randomization to either the intervention or control arm will occur independently for each module. That is, group allocation for a module does not predetermine that allocation for subsequent modules. Randomization will be stratified by team size (small or large) and location (west, central, or east) to ensure balanced sub-groups. Randomization is performed independently by using a computer-generated sequence. The randomization procedure will be centralized and managed by an independent statistician.

### Data analysis

The unit of analysis will be the PCP team. Survey (quantitative) data will be used to compare treatment groups with respect to team and individual characteristics (potential covariates) by tabulation methods (means, standard deviations, frequencies). Both team level variables (e.g., region) and member level variables (e.g., palliative care training) will be tabulated. The primary analysis will be a between treatment comparison of intervention and control groups of the change in pre versus post module scores on the primary outcomes (EPCS and % identified for palliative care). Secondary analyses will include comparisons of all team outcomes. Mixed model ANOVA methods will be used, taking into account the increased variance due to cluster randomization, for the assessment of the primary outcomes [30]. Multilevel mixed models with two levels, cluster and repeated measures will be used to investigate the effect of the intervention over time (baseline, post module, 6 months post module).

#### Qualitative analysis (focus group data)

We will conduct a thematic analysis using a constant comparison method along a 4-stage process based on Pope’s Framework Approach [31], as we have done previously [32, 33]: 1) Focus groups will be audio taped and transcribed into a document, along with staff notes, for analysis. 2) The Focus group questions will be used to create a template for organizing each team’s data and emerging ideas. 3) Emerging ideas from each template construct will be coded and compared within and across teams, first independently by two analysts and then conjointly. Emerging themes will be compared and discussed until consensus is obtained between the analysts. 4) Common themes for each construct will be identified. We will maintain an audit trail that documents and justifies decisions in the analysis to promote consistency [34].

#### Evaluative framework

We will use the Kirkpatrick Model, a globally recognized training evaluation framework, to frame the various program evaluation components (Table 2). This model outlines 4 critical domains of an effective training program [35]:

- Reaction: The participant’s reaction or satisfaction to the education program.
- Learning: The participant’s acquired knowledge and skills from the education program.
- Behaviour: The participant’s application of what they learned during the program to their practice.
- Results: The direct outcomes, e.g., patient outcomes, that occur as a result of the education program.

## Discussion

This study protocol details the implementation and evaluation of CAPACITI, a training intervention for guiding generalist health care providers to operationalize a palliative approach to care in primary care practice. The findings from this large-scale, national cRCT will contribute to the evidence base on how to strengthen the ability of primary care physicians, nurses, and other providers to identify and manage their patients requiring palliative care, and in turn, build capacity for this care in the health care system. In particular, we will assess the utility of a facilitated approach to training education. CAPACITI was developed on the premise that context-relevant practice supports are vital to impacting provider behavior, based on knowledge translation science showing that education or tools alone do not change practice without context-relevant information [36–38].

One of the features of this intervention is that it aims to build capacity among interprofessional primary care providers in the community. Doing so does not preclude the need for specialist palliative care in the community but rather recognizes that not all patient needs require secondary level palliative care by specialist teams [3, 4, 39, 40] As such, the program strengthens community-based palliative care capacity within the existing team and health system, capitalizing upon existing expertise, without the need for new front-line human resources or the implementation of formalized system structures [18]. Thus, any built-capacity is more likely to be sustained beyond the program.

Our CAPACITI trial will help address a lack of high-level evidence on knowledge and behavior change in palliative care education for generalist providers using a virtual format with case-based, facilitation. A recent systematic review of trials identified 22 palliative care education interventions for health care providers [21]. Many of these initiatives focused on illness communication or symptom management rather than a wider array of skills to implement a holistic palliative approach to care. As well, most studies focused on a single provider type rather than an interdisciplinary team. Overall, published reviews have concluded that while interactive education inventions for health care providers show potential for building palliative care capacity among generalists, further trial-based evidence is required, especially among virtually delivered programs [41–44].

The proposed CAPACITI cRCT has several conceptual and methodological strengths to be considered. Many previous randomized trials on palliative care education had small sample sizes (20 to 40 per arm) and/or selected participants from a single site [21]. Whereas we expect to enroll a relatively large number of participants, over 200 PCPs per arm, from teams across Canada. This will help ensure that our study has sufficient analytical power, and that the findings are generalizable, at least to other locales in this country. We have designed the program to be virtual (materials and discussions) so that it is scalable, accessible, and cost-effective to implement. The learning management system (Moodle) will allow all participants in the trial to have access to the education materials and enable us to deliver the program to the anticipated 100 + teams that enrol. Moreover, the virtual platform allows teams and providers in rural and remote areas to access the program, overcoming the barriers of travel and geography.

### Limitations

This study has some foreseeable limitations deserving mention. Both study arms will have access to the CAPACITI education materials and therefore we will not have a randomized group that receives “no-CAPACITI education” to compare. PCPs who sign up to the program but receive no education would be unlikely to complete the study measures. Previous RCTs have found non-interactive or non-facilitated on-line palliative care training modules to be effective at improving Palliative care knowledge [45] or attitudes [46] in palliative care. Therefore, it is possible that both arms in the current study will experience a positive effect, the difference which may be negatable between arms, post intervention. Secondly, as typical with RCTs of training interventions, participants will be aware which study arm they have been allocated. We deemed it unfeasible to blind participants to the group arms. The use of self-reported measures presents another limitation as these may be biased toward behaviour change. More objective measures of practice change, such as patient outcomes and administrative data, are warranted but obtaining these data present challenges without existing systems that collect this information. The impact of patient reported outcomes and health service utilization at end of life could be examined in future research. To help validate the quantitative outcomes we will explore the impact of CAPACITI from a qualitative perspective of the PCPs.

## Conclusions

CAPACITI is a unique education program for generalist PCPs in that this intervention is interdisciplinary and comprehensive, as well as virtual. Through this cRCT we will ascertain the added benefit of a facilitated approach to the delivery of education materials, towards informing the most efficient means of translating this knowledge to practice among PCPs and other health care providers. The findings from this health provider education trial may also be applicable to non palliative care specialists and health care providers in tertiary settings.

## Supplementary Information

**Additional file 1.**

## Data Availability

This is a study protocol from which no data are available.

## Abbreviation

PCP: Primary Care Provider
