# Supplementary material for: Increasing palliative care capacity in primary care: study protocol of a cluster randomized controlled trial of the CAPACITI training program
Source: BMC Palliat Care. 2023 Jan 5;22:2. doi: 10.1186/s12904-022-01124-x (PMC9813458; doi:10.1186/s12904-022-01124-x)
Supplement: Supplementary file 1 — Additional file 1. [file 12904_2022_1124_MOESM1_ESM.pdf]

CAPACITI program

Supporting primary care teams to provide early palliative care

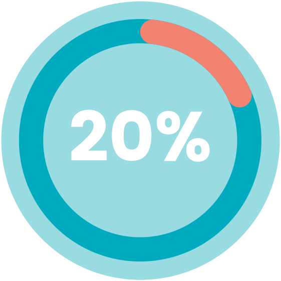

- **>80% of patients could benefit from palliative care, but only 20% get home-based palliative care**
- Palliative care is not only for end of life, but should begin earlier in the disease trajectory
- Many primary care teams want to provide early palliative care, but want practical tips on how

- **CAPACITI is an education program for interprofessional primary care teams**
- Provides practical tips and strategies from national experts
- Shows how to incorporate an early palliative care approach into practice
- There are 3 modules organized into 3 cohorts

How the CAPACITI program can help

1. Identify & Assess Like an Expert
2. Enhance Communication Skills
3. Enhance Skills for Ongoing Care

DETAILS

- All teams will receive videos & materials. Half the teams will be randomized to also receive virtual facilitation.
- Time commitment: 1 module is 4 hours over 2 months.
- Costs: As a CIHR-research project, the program is free.
- Modules are offered once per cohort.

BENEFITS

- Increased knowledge
- More job satisfaction
- More proactive practice
- Increased collaboration
- Increased confidence

Who should apply?

- **Generalist primary care providers (not palliative care specialists) who want to embed palliative care approaches into their practice.**
- We are encouraging primary care teams* to apply.
- Teams from anywhere across Canada can join.

* E.g. Teams are physicians, nurse practitioners, nurses, social workers, main office assistant, complex patient coordinator, etc. -- anyone involved in the care of a patient with serious illness within a practice.

CAPACITI program

Supporting primary care teams to provide early palliative care

Schedule

Here is the schedule of modules to participate in wave 2 of CAPACITI

	COHORT 1	COHORT 2
1. Identify & Assess Like an Expert		
2. Enhance Communication Skills		
3. Enhance Skills for Ongoing Care		

Please note: Each module is 4 unique bi-weekly sessions over 2 months.

This project has been reviewed by the Hamilton Integrated Research Ethics Board under study number #13867

Need more info?

1. **Information sessions (hosted with Pallium Canada)**
   - Coming soon in January & February 2022
2. **Website**
   - Registration for information sessions
   - More information about the program
   - Officially sign-up for CAPACITI
3. **More questions?**
   - Contact us at xxxxx

Project ECHO Palliative Care - Canada BY Pallium Canada

www.palliativecareinnovation.com

Ver 2, 27.10.2021

PALLIATIVE CARE INNOVATION
